# The Role of Country Image on Consumers’ Willingness to Pay for Imported Beefsteak in China

**DOI:** 10.3390/foods13060938

**Published:** 2024-03-20

**Authors:** Erpeng Wang, Mingyuan Ji, Lingyu Wang, Yuefeng Wu, Zeyu Shi

**Affiliations:** School of Economics and Management, Nanjing Tech University, Nanjing 211816, China

**Keywords:** country image, willingness to pay, imported beefsteak, political conflict, consumer perception

## Abstract

In recent years, the world has experienced conflict. When political conflicts affect consumers’ emotions and alter their perceptions of a country’s image, it can influence their preferences. This study deconstructs the notion of a country image into multiple dimensions and examines their impact on consumers’ willingness to pay for imported beef from Australia, Brazil, and the United States. Using a rank-ordered probit model and data from a survey of 935 respondents, results show that consumers’ perceptions of a country’s friendliness, economy, environment, and quality all have a positive and statistically significant effect on their willingness to pay for beefsteak imported from that country. Among these dimensions of the country image, the quality is the most important, followed by the economy, friendliness, and the environment. This study also finds heterogeneity in consumer perception of friendliness towards the United States, Australia, and Brazil. This study provides valuable insights for assessing the real losses resulting from a deteriorating international environment and suggests policies to enhance competitiveness in the food market.

## 1. Introduction

In recent years, the world has experienced numerous conflicts, including strained EU–Russia relations, tensions between China and the United States, and deteriorating China–Australia relations. Furthermore, modern social media increasingly plays a role in conflict and contentious politics. Social media not only provides an important resource but also fundamentally changes the information available to conflict actors, thereby shaping the conflict itself [1]. This can affect consumers’ emotions in these countries, thereby altering their perceptions of the country image. Roth and Romeo [2] claimed that if a country is perceived as having a positive image and that image is important to a product category, consumers will be more willing to buy products from that country. But what if political conflict hurts consumers’ emotions and changes their perceptions of these countries? Would this influence consumer preferences and inevitably impact bilateral economic and trade ties? A deeper understanding of the effect of the country image could provide policymakers and agribusinesses with valuable information for assessing the economic losses resulting from political conflict. This is especially important in a world where social media can amplify the effects of political conflict.

In the food market, the country of origin (COO) provides important information to assist consumers in their decision-making process [3]. Previous research on consumer preferences for beef with COO labeling has found a strong preference for domestic beef [4,5,6,7]. Chinese consumers have considered the country of origin to be an important factor in their purchasing decisions for milk products [8,9,10]. The country of origin is an important signal of the quality and safety of food products and serves as a valuable informational cue guiding consumer behavior when the product information is asymmetric [11,12]. The country of origin invokes consumers’ knowledge and beliefs about the place of production [13,14,15,16]. Consumers may derive perceptions about the safety and quality of food products from the country of origin [13,16]. Perceived quality differences between domestic and foreign-made products can induce consumers to prefer imported goods if they are perceived as being of higher quality [17]. In an ideal setting, the country of origin labeling (COOL) enables consumers to make informed purchasing decisions by providing them with information that helps them choose products that best fit their preferences.

However, whether the country of origin labeling (COOL) on food can achieve its intended policy outcome depends on consumers’ image of the country. The country image refers to the mental representation of a country and its people, including cognitive beliefs about its economic and technological development stages, as well as affective evaluations of its social and political systems or standpoints [18]. Numerous studies have investigated whether consumers develop certain images of countries of origin and how those images influence their acceptance of foreign-made goods and services [19,20]. Furthermore, while previous studies have often measured the country image by a country’s economic and technological level, few have considered the effect of consumers’ emotions towards the country. Consumers’ preferences have been shown to be affected by their ethnocentrism [21] and patriotic emotions can influence their perceptions and choices of domestic products [22,23]. For example, despite most Chinese appreciating Japan as a creative and highly developed country, only one in five (21%) held favorable views of Japan due to its invasion of China during World War II [24]. While studies have shown that Chinese consumers prefer imported food [25,26], the motivation for such preferences is not well explored in the literature [15]. Therefore, it is urgent to study how consumers’ emotional dimension of a country image affects their willingness to pay for imported food in light of increasing political conflict in the world.

The objective of this study was to decompose the construct of the country image (CI) into multiple dimensions and to investigate how consumers’ country images, particularly the emotional dimension, affects their willingness to pay for imported beef. The results of this study contribute to the existing literature by introducing the CI construct and its multiple dimensions to gain in-depth knowledge about assessing the real losses resulting from a deteriorating international environment. 

## 2. Literature Review

The country image is often defined as a consumer’s perception of an origin country’s people, development standards, similarity to their own country, and the general quality of products made in that origin country [17]. Country image or country-of-origin effect studies have become a significant and popular area of international business research in recent decades [18]. The image of a country can be partially formed through prior experience with products from that country [27]. Some previous studies found that consumers tend to give higher evaluations to food products originating from industrialized or economically developed countries [28,29,30].

Some marketing research has focused on the complex construct of a country image by decomposing the effects of COO into dimensions at the macro level related to the country image, and four dimensions at the micro-level related to product attributes [26]. These studies analyze the COO effect on consumers’ perceptions of a country image [31,32]. While the image of a country can be partially formed through prior experience with products from that country, a country’s general image is somewhat distinct from products associated with a particular country [27]. While previous studies have emphasized the effect of the country image in different dimensions [26,33], especially in the domain of durable goods, there has been little related research in the field of food economics [26]. A key question in this area of research is whether the country image actually influences product evaluation and purchase [34]. In particular, the cognitive country image is perceived to influence the product image associated with a country, which acts as an important cue that influences perceived product quality and leads to purchase intention [18].

Although individuals often have stereotypical beliefs about particular attributes associated with the product image of certain countries, their valuation of products may change due to a collapse in the country’s image. Even past positive country images can be ruined by events that hurt consumers’ emotions, especially in light of increasing political conflict in the world. Some consumers may reject products labeled as imports due to their ethnocentrism or patriotism. Numerous publications in the marketing literature have examined how consumer ethnocentrism or patriotism affects their choice of domestic versus imported goods [35,36].

While previous studies have emphasized the effect of the country image in different dimensions [33], especially in the domain of durable goods, there has been little related research in the field of food economics [26]. Previous years’ studies have shown that Chinese consumers prefer imported food [26], but the motivation for such preferences is not well explored in the literature [15]. Policymakers are eager to know the impact of political conflict on country images, thereby affecting consumers’ preferences. It is therefore urgent to study how consumers’ country images affect their willingness to pay for imported products in light of increasing political conflict in the world. A deeper understanding of the effect of the country image could provide policymakers and agribusinesses with valuable information for assessing the losses resulting from political conflict. 

## 3. Research Method

### 3.1. Survey Design

This study uses beefsteaks as an example to examine consumers’ country images and preferences in the Chinese food market. China is the second-largest consumer of beef, with its consumption having increased by 51% from 2010 to reach 9.5 million tons in 2020 (What beefed up US exports to China could mean for Australian cattle prices, https://www.ruralbank.com.au/blog/knowledge-and-insights/what-beefed-up-us-exports-to-china-could-mean-for-australian-cattle-prices/ (accessed on 12 May 2023)). This study focuses on the three main countries that export beef to China—Australia, Brazil, and the United States—as they are ranked as the top three major exporters to China. Australia is one of the largest beef exporters to China, having exported USD 3 billion worth of red meat, including beef, to China in 2020. The United States is the world’s largest beef producer and recently began exporting beef to China after a ban lasting for more than a decade. In 2021, the United States exported USD 1.6 billion worth of beef to China (Beef and Beef Products 2021 Export Highlights, https://fas.usda.gov/beef-2021-export-highlights (accessed on 6 March 2024)). Brazil is China’s top beef export provider, having exported around USD 4 billion worth of beef to China in 2020, accounting for 48 percent of its total exports (China lifts embargo on Brazilian beef, https://www.france24.com/en/live-news/20211215-china-lifts-embargo-on-brazilian-beef (accessed on 12 May 2023)).

The country image is often referred to in a general sense and mainly focuses on the economic, technological, social, and political variables of a country [37,38]. There are no unique scales available to accurately measure the abstract concept of a country image. In this study, we used some of the validated scales from the country image literature to assess consumers’ country images. We decomposed the country image into four dimensions and used a total of four statements in the survey (Table 1). The first three items were validated scales measuring the general country image, adapted from Parameswaran and Pisharodi [39], while the last statement pertained to consumers’ feelings about the country. To measure the country image, the participants were asked to indicate their level of agreement with statements such as “Country X has a high level of technological research” on a scale of 1 (strongly disagree) to 7 (strongly agree) for each of the three countries (Australia, United States, Brazil). Furthermore, these countries are especially renowned for their rich endowment of water and grassland resources, which certify the quality of their dairy products. The last item is the product image, which refers to consumers’ general perceptions of a particular country’s products [39] or their total beliefs regarding products from a given country [40].

Contingent valuation (CV) is a popular stated preference method used to elicit consumer preferences for market and non-market goods [41,42]. It is used to measure consumer willingness to pay (WTP). In this study, we used the payment card approach which is prevalent in the current literature [43]. The WTP values estimated by a payment card approach are more robust than those relying on a direct choice approach [44]. Respondents were asked to choose the one value which represents their maximum WTP values [45]. The true WTP of the respondents was then assumed to be located above the indicated value and below the next higher one, if such a value exists [46]. In the survey, we requested respondents to pick the maximum acceptable price for a piece of beefsteak imported from XXX country. We set the choices of prices in the payment card including five intervals: CNY 6–12, CNY 12–18, CNY 18–24, CNY 24–30, and CNY 30 or more. Hence, hypothetical bias stems from the lack of incentive compatibility in the experiment, which may lead to an overstatement of the WTP. In the following analysis, respondents’ willingness to pay was also ranged to be in order.

### 3.2. Data Collection

Data for our online survey were collected from Beijing, Shanghai, and the provinces of Jiangsu, Guangdong, Shandong, Henan, and Inner Mongolia. Beijing is China’s capital and has a population of over 21 million. Shanghai is a global financial, research, technology, manufacturing, and transportation hub and the third most populous city in the world. Both cities had a GDP per capita exceeding CNY 150,000 in 2020. Jiangsu and Guangdong are developed coastal provinces and major beefsteak consumption areas. Jiangsu and Guangdong are the two provinces with the highest gross domestic product (GDP) in China. Specifically, Guangdong and Jiangsu led the list with GDPs of CNY 12.9 trillion and CNY 12.3 trillion, respectively. Shandong, Henan, and Inner Mongolia are known for beef production and consumption. Henan and Shandong are major beef cattle farming regions in agricultural areas, while Inner Mongolia is a major beef cattle breeding region in pastoral areas [47], with a production of 663,000 tonnes of beef in 2020 (Inner Mongolia’s beef output ranks first in China, http://www.news.cn/english/2021-08/26/c_1310150281.htm (accessed on 6 March 2024)).

The online survey was conducted by a professional marketing research company (www.wjx.cn), which closely monitored the sampling process to ensure sample quality. Several measures were taken to this end. First, respondents were randomly selected from a sample library of 2.6 million people. They were asked to answer questions about their income, family size, and job that they had previously answered. Only those providing consistent answers could proceed with the survey. Second, the company ensured that each questionnaire was completed by a unique I.P., computer, and account. Third, respondents had to meet specific requirements such as being at least 18 years old and being online beefsteak buyers. Finally, the “trap question” method [48,49] was used to identify respondents who may not have carefully read the survey questions. We received a total of 2008 responses. After excluding those who failed to meet all four criteria, 935 respondents were included in the final analysis.

### 3.3. Econometric Model

A rank-ordered probit model was used to explore the impact of the country image on consumers’ willingness to pay for imported beefsteak. A rank-ordered probit model is a type of econometric model that is used to analyze data from rank-ordered choices [50]. This model allows us to relax the independence of irrelevant alternatives (IIA) property that is characteristic of the rank-ordered logistic model by estimating covariances between the error terms for the alternatives. The model is fitted using maximum simulated likelihood. It has been applied in various studies to understand preferences for alternative options [51].

Consider the utility of a J alternative rank-ordered probit model: we have utilities (latent variables) $U_{ij},j=1,\ldots ,J$, such that

$$U_{ij}=x_{ij}\beta +z_{i}\alpha_{j}+\epsilon_{ij}$$

Here, $x_{ij}$ are the alternative-specific independent variables, which refer to the country image dimensions including friendliness, economy, environment, and quality; $z_{i}$ are the case-specific variables, including respondents’ characteristics, and $\epsilon_{ij}$ are multivariate normal variables with mean zero and covariance Ω.

Without loss of generality, assume that individual i ranks the alternatives in order of alternative indices j=1,…,J, so the alternative J is the preferred alternative, and alternative 1 is the least preferred alternative. The probability of this ranking given β and $\alpha_{j}$ is the probability that $U_{i,J-1}-U_{i,J}\leq 0$ and $U_{i,J-2}-U_{i,J-1}\leq 0,\ldots ,$ and $U_{i,1}-U_{i,2}\;\leq 0$. In this study, the willingness to pay (WTP) is treated as an ordered variable, with higher values indicating a preference for the alternative. We used the Comprobit command in Stata 16 to fit rank-ordered probit models using maximum simulated likelihood, which allows us to relax the assumption of independence. 

## 4. Empirical Results

### 4.1. Data and Descriptive Statistics

Table 2 presents a summary of the statistics. The majority of respondents were female (56.36%), had 13–16 years of education (86.31%), and came from families with at least one child under the age of 12 (67.49%). The average age of respondents was about 30 years old, and the median household monthly income was between CNY 16,000 and 20,000. The income varied among study areas, with respondents in Beijing and Shanghai reporting the highest monthly income and those in Henan and Inner Mongolia reporting the lowest. Our sample had a higher proportion of young, female consumers with higher education and income levels because they were more likely to buy fresh agricultural products online. In addition, about 54% of respondents reported purchasing beefsteak online sometimes, while 46% reported doing so very often. 

Table 3 shows Chinese consumers’ perceptions of the country images of Australia, America, and Brazil. Half of the respondents agree or strongly agree that Brazil is friendly towards them, a slightly higher percentage than that of Australia. In contrast, less than 10% of respondents have the same perception of America. Furthermore, nearly 60% of respondents strongly disagree or disagree that America is friendly towards them, followed by Austria. For Brazil, less than 10% of respondents strongly disagree or disagree that it is friendly towards them. Overall, respondents feel more hostility from America and friendlier towards Brazil, with a significant segment for Australia.

**Figure 1:**
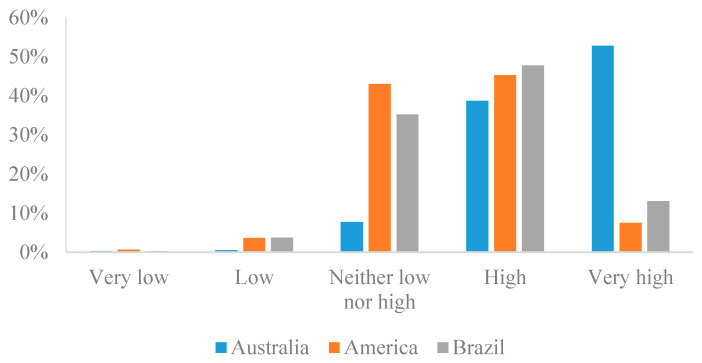
Consumers’ quality perception of beefsteak from different countries.

Figure 1 shows respondents’ quality perception of beefsteak from different countries. According to the figure, 52.73% of respondents rated the quality of beefsteak from Australia as excellent, while only 7.49% and 13.05% rated the quality of beefsteak from America and Brazil as excellent, respectively. Conversely, 0.64% and 0.32% of respondents rated the quality of beefsteak from America and Brazil as very low, while only 0.32% rated the quality of beefsteak from Australia as very low.

**Figure 2:**
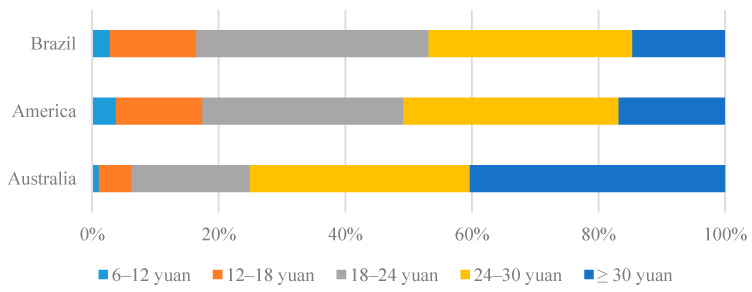
Consumers’ willingness to pay for beefsteak from different countries.

As shown in Figure 2, there are differences in Chinese consumers’ willingness to pay for beefsteak imported from Australia, America, and Brazil. Approximately 40% of respondents are willing to pay more than CNY 30 for a piece of beefsteak imported from Australia, while less than 20% are willing to pay the same amount for beefsteak imported from Brazil or America. To calculate the mean willingness to pay (WTP), we assumed that the true value is the midpoint of the interval. For the upper-open interval, we assumed that the WTP value is equal to the sum of the lower boundary and half the distance to the neighboring interval. The mean WTP values for a piece of beefsteak imported from Australia, America, and Brazil are CNY 27.706, CNY 24.350, and CNY 24.298, respectively.

### 4.2. Econometric Results

Table 4 presents the results of a rank-ordered probit model that estimates the WTP for beefsteak imported from Australia and Brazil, taking America as a base. The results show that perceptions of friendliness, economy, environment, and quality all have a positive and statistically significant effect on the WTP. The coefficient for friendliness is 0.168, indicating that consumers are willing to pay more for beefsteak imported from countries that they perceive as friendly. The coefficient for economy is 0.268, which shows that consumers are willing to pay more for beefsteak imported from countries with strong economies. Similarly, a one-unit increase in the perception of a country’s environment and quality is associated with an increase of 0.161 and 1.262 in the WTP, respectively. In contrast, the demographic characteristics included in the model do not have a statistically significant effect on the WTP. Overall, these results suggest that consumers’ perceptions of a country’s friendliness, economy, environment, and quality are important determinants of their willingness to pay for beefsteak imported from that country.

We further calculated the relative importance (RI) of each dimension of the country image in influencing Chinese consumers’ WTP for imported beefsteak. We calculate the RI of a dimension by dividing its range of utility change estimates by the sum of the ranges for all dimensions and multiplying by 100. Applying this formula to the coefficients from Table 4, we found that quality is the most important dimension, with a relative importance of 67.89%, followed by economy (14.41%), friendliness (9.04%), and environment (8.66%). 

Furthermore, an ordered logit model was used to analyze heterogeneity in consumer perception of friendliness towards the United States, Australia, and Brazil. The results in Table 5 show that being female has a positive effect on the perception of friendliness towards the United States and Australia, but not towards Brazil. Education level has a positive effect on the perception of friendliness towards the United States, but not towards Australia or Brazil. Consumption level has a positive effect on the perception of friendliness towards all three countries, implying that as consumption level increases, consumers are more likely to perceive these countries as friendly. Respondents who have children under 12 years old have a positive perception of friendliness towards the United States and Brazil, but not towards Australia.

## 5. Discussion and Conclusions

In recent years, the world has experienced conflict. While a large body of literature has studied the direct impact of tariffs and trade barriers on national trade, there has been relatively little research on how political conflicts can affect consumers’ emotions and alter their perceptions of a country’s image, ultimately influencing their preferences. This study addresses this gap by examining the case of imported beefsteak. By decomposing the concept of a country image into multiple dimensions, this study investigates how consumers’ emotions impact their willingness to pay for imported beef from Australia, Brazil, and America. The findings provide vital information for evaluating the indirect loss resulting from conflict between countries.

The results from our statistical analysis show that there are differences in Chinese consumers’ perceptions of the country images of Australia, America, and Brazil. Respondents feel more hostility from America and friendlier towards Brazil, with a significant segment for Australia. This is reflected in their willingness to pay for beefsteak imported from these countries. Approximately 40% of respondents are willing to pay more than CNY 30 for a piece of beefsteak imported from Australia, while less than 20% are willing to pay the same amount for beefsteak imported from Brazil or America. The mean willingness to pay values for a piece of beefsteak imported from Australia, America, and Brazil are CNY 27.706, CNY 24.350, and CNY 24.298, respectively.

The results from our rank-ordered probit model show that consumers’ perceptions of a country’s friendliness, economy, environment, and quality all have a positive and statistically significant effect on their willingness to pay for beefsteak imported from that country. Among these dimensions, quality is the most important, followed by economy, friendliness, and environment. These findings are consistent with previous studies that have emphasized the effect of the country image on consumers’ valuation of imported products and purchase intention [18,33]. This mechanism is very similar to geographical indications [52,53]. Furthermore, the study found heterogeneity in consumer perception of friendliness towards the United States, Australia, and Brazil. Female respondents have a positive perception of friendliness towards the United States and Australia but not towards Brazil. The education level has a positive effect on the perception of friendliness towards the United States but not towards Australia or Brazil. The consumption level has a positive effect on the perception of friendliness towards all three countries. 

In conclusion, this study provides important insights into how country images affect Chinese consumers’ willingness to pay for imported food products. The results suggest that improving a country’s image in terms of friendliness, economy, environment, and quality can increase its exports of food products to China. These findings have important implications for policymakers and agribusinesses seeking to assess the economic loss of political conflict and improve their competitiveness in the Chinese food market. This approach provides a deeper understanding of the effect of the country image on consumer preferences and can help policymakers and agribusinesses assess the loss resulting from political conflict. The results from this study can provide important information to assess the real loss of the deteriorating international environment.

Based on the fundamental principles of economics, trade can make everyone better off. However, the ongoing increase in political conflicts has already affected consumers’ emotions and altered their perceptions and preferences, leading to severe losses in human welfare. Our understanding of how political conflict harms consumers’ emotions, influences consumer preferences, and inevitably impacts international trade, remains limited. Future research could further explore the underlying mechanisms through which the country image affects consumer preferences for imported food products. For example, researchers could investigate how social media influences consumers’ perceptions of country images and how this in turn affects their willingness to pay for imported food products. Additionally, future research could examine how consumers’ feelings towards a country change over time in response to political events and how this affects their preferences for imported food products.

## Figures and Tables

**Table 1:** Measurement items of country images.

Categories	In Your Perception, Country XXX	Australia	America	Brazil
General country image	Is friendly to us	1 = Strongly disagree, 7 = strongly agree		
	Is economically well-developed			
	Has a good natural environment			
Product image	Quality perception of beefsteak imported from XXX country	1 = Very low quality, 5 = very high quality		

**Table 2:** Comparison of demographic and behavioral characteristics for participants from different areas.

Sample Variable	Beijing (N = 140)	Shanghai (N = 212)	Jiangsu (N = 173)	Shandong (N = 112)	Guangdong (N = 153)	Henan (N = 123)	Inner Mongolia (N = 22)	Pooled Sample (N = 935)
**Gender:**								
Male	39.29	39.15	52.6	41.07	40.52	47.15	59.09	43.64
Female	60.71	60.85	47.4	58.93	59.48	52.85	40.91	56.36
**Age:**	30.61	32.35	30.87	31.45	29.92	28.03	30.91	30.71
**Education level:**								
≤12 years	0.71	2.36	1.73	2.68	3.27	0.00	0.00	1.82
13–16 years	87.86	86.32	87.86	75.89	90.85	86.99	81.82	86.31
>16 years	11.43	11.32	10.4	21.43	5.88	13.01	18.18	11.87
**Family monthly income** **:**								
CNY <8000	5.71	2.83	6.94	8.93	11.76	12.20	9.09	7.59
CNY 8000–12,000	12.14	8.02	16.18	25.00	12.42	26.02	31.82	15.83
CNY 12,000–16,000	17.14	14.62	23.12	24.11	22.88	21.95	13.64	20.00
CNY 16,000–20,000	25.71	26.89	21.97	21.43	26.80	17.07	22.73	23.74
CNY 20,000–40,000	30.71	42.45	28.90	16.96	22.22	18.70	18.18	28.13
CNY ≥40,000	8.57	5.19	2.89	3.57	3.92	4.07	4.55	4.71
**Children under 12 years old:**								
No	37.86	39.62	35.26	23.21	32.03	19.51	31.82	32.51
Yes	62.14	60.38	64.74	76.79	67.97	80.49	68.18	67.49
**Consumption level** ^a^**:**								
CNY 6–12/150 g	0.71	0.94			1.96	1.63		0.86
CNY 12–18/150 g	12.86	9.43	16.18	11.61	7.84	12.20	18.18	11.76
CNY 18–24/150 g	30.71	30.66	29.48	29.46	31.37	20.33	27.27	28.98
CNY 24–30/150 g	34.29	32.55	31.79	31.25	30.72	29.27	27.27	31.66
CNY ≥30/150 g	21.43	26.42	22.54	27.68	28.10	36.59	27.27	26.74

Note: ^a^ the price that respondents usually pay for a piece of beefsteak online.

**Table 3:** Country image of different countries.

In Your Perception,	Strongly Disagree	Disagree	Neither Agree Nor Disagree	Agree	Strongly Agree
Australia:					
Australia is friendly to us	6.95	13.80	29.52	39.25	10.48
Australia is economically well developed	0.64	3.10	21.28	50.80	24.17
Australia has a good natural environment	0.43	1.60	7.70	37.97	52.30
America:					
America is friendly to us	22.99	36.04	32.51	7.70	0.75
America is economically well developed	0.53	0.86	5.78	44.92	47.91
America has a good natural environment	2.35	11.23	42.25	37.65	6.52
Brazil:					
Brazil is friendly to us	1.28	6.10	35.83	48.24	8.56
Brazil is economically well developed	3.74	29.63	46.31	16.36	3.96
Brazil has a good natural environment	1.93	5.78	29.95	44.39	17.97

**Table 4:** Rank-ordered probit model parameter estimates.

WTP	Coef.	Std. Err.	z	P > z	Interval	
Country Image						
Friendly	0.168	0.065	2.58	0.010	0.040	0.295
Economy	0.268	0.064	4.18	0.000	0.142	0.393
Environment	0.161	0.065	2.46	0.014	0.033	0.289
Quality	1.262	0.100	12.6	0.000	1.066	1.459
Australia						
Female	−0.084	0.199	−0.42	0.674	−0.473	0.306
Age	0.004	0.013	0.29	0.774	−0.022	0.030
Edu	−0.296	0.258	−1.15	0.250	−0.802	0.209
Consumption_level	−0.151	0.100	−1.51	0.130	−0.347	0.045
Intercept	1.622	0.803	2.02	0.044	0.047	3.196
Brazil						
Female	−0.014	0.172	−0.08	0.937	−0.352	0.324
Age	−0.007	0.011	−0.64	0.524	−0.029	0.015
Edu	−0.038	0.225	−0.17	0.865	−0.479	0.403
Consumption_level	−0.109	0.084	−1.3	0.195	−0.273	0.055
Intercept	0.581	0.722	0.8	0.421	−0.835	1.997

**Table 5:** Ordered logit model parameter estimates of the perception of friendliness.

Variable	America	Australia	Brazil
Female	0.438 ***	1.045 ***	0.0655
	(0.124)	(0.127)	(0.127)
Age	−0.00266	−0.00736	−0.00895
	(0.00852)	(0.00847)	(0.00859)
Education	0.281 *	−0.0125	0.0853
	(0.169)	(0.167)	(0.178)
Consumption level	0.142 **	0.236 ***	0.240 ***
	(0.0601)	(0.0600)	(0.0614)
Child	0.238 *	0.175	0.296 **
	(0.131)	(0.131)	(0.135)

Note: * Indicates statistically significant at the 10% significance level; ** indicates statistically significant at the 5% significance level; *** indicates statistically significant at the 1% significance level.

## Data Availability

The original contributions presented in the study are included in the article, further inquiries can be directed to the corresponding author.
